# How Hives Collapse: Allee Effects, Ecological Resilience, and the Honey Bee

**DOI:** 10.1371/journal.pone.0150055

**Published:** 2016-02-24

**Authors:** Brian Dennis, William P. Kemp

**Affiliations:** 1 Department of Fish and Wildlife Sciences, University of Idaho, Moscow, Idaho, 83844–1136, United States of America; 2 Department of Statistical Science, University of Idaho, Moscow, Idaho, 83844–1136, United States of America; 3 USDA Agricultural Research Service, Red River Valley Agricultural Research Center, 1605 Albrecht Blvd North, Fargo, North Dakota, 58102–2765, United States of America; University of Sheffield, UNITED KINGDOM

## Abstract

We construct a mathematical model to quantify the loss of resilience in collapsing honey bee colonies due to the presence of a strong Allee effect. In the model, recruitment and mortality of adult bees have substantial social components, with recruitment enhanced and mortality reduced by additional adult bee numbers. The result is an Allee effect, a net per-individual rate of hive increase that increases as a function of adult bee numbers. The Allee effect creates a critical minimum size in adult bee numbers, below which mortality is greater than recruitment, with ensuing loss of viability of the hive. Under ordinary and favorable environmental circumstances, the critical size is low, and hives remain large, sending off viably-sized swarms (naturally or through beekeeping management) when hive numbers approach an upper stable equilibrium size (carrying capacity). However, both the lower critical size and the upper stable size depend on many parameters related to demographic rates and their enhancement by bee sociality. Any environmental factors that increase mortality, decrease recruitment, or interfere with the social moderation of these rates has the effect of exacerbating the Allee effect by increasing the lower critical size and substantially decreasing the upper stable size. As well, the basin of attraction to the upper stable size, defined by the model potential function, becomes narrower and shallower, indicating the loss of resilience as the hive becomes subjected to increased risk of falling below the critical size. Environmental effects of greater severity can cause the two equilibria to merge and the basin of attraction to the upper stable size to disappear, resulting in collapse of the hive from any initial size. The model suggests that multiple proximate causes, among them pesticides, mites, pathogens, and climate change, working singly or in combinations, could trigger hive collapse.

## Introduction

Honey bees (*Apis mellifera*), in addition to generating a wide range of hive products for human consumption, provide irreplaceable pollination services to agricultural and natural ecosystems. Although the domestication of the honey bee is closely connected to the evolution of food-based socio-economic systems in many cultures throughout the world [1], in current economic terms, and in the U.S. alone, the estimated wholesale value of honey, more than $320 million dollars in 2014 [2], pales in comparison to aggregate estimated annual value of pollination services, variously valued at $11–15 billion [3].

Yet, it is generally agreed that domestic and feral honey bees in North America are in crisis due to a variety of stressors including pathogens, parasites, the rigors of migratory beekeeping associated with pollination of crops like California almonds, as well as environmental factors such an forage availability and xenobiotics [4, 5, 6, 7, 8, 9, 10]. Additionally, one third to one half of the 30% average annual wintering losses experienced by self-reporting U.S. beekeepers has been associated with the phenomenon known as Colony Collapse Disorder [11, 12]. CCD is characterized by the mysterious loss of *hive-level* viability and is widespread.

Although numerous inimical factors have been implicated, no single cause for CCD has gained consensus. Authors of the first comprehensive survey of CCD-affected honey bee colonies [13] considered no fewer than 61 quantifiable variables. The authors ultimately could only associate the occurrence of CCD events (localized aggregations of failed hives) to interactions between “pathogens and other stress factors.” Furthermore, extensive literature reviews [4, 5], while adding useful context to the complex nature of the CCD phenomenon, fail to provide a coherent narrative beyond the apparently singular contribution of *Varroa* mites (specifically, *Varroa destructor*) as an associated factor, due either to direct parasitism, the mite’s role in vectoring a wide range of potentially debilitating diseases, or the compounding and associated stress to honey bees of in-hive control measures required for *Varroa* during the late season declines in hive-level reproductive output.

It is important to note that the CCD phenomenon that beekeepers and researchers are attempting to describe is measured at the hive or population level. Under management and in nature, honey bee colonies, or hives, either survive or they fail for a variety of reasons [11]. It is also important to note that, each hive, or normally functioning population, has only one queen. Thus, regional “population dynamics” of colonies managed in aggregate—the sum of the potential pollination services potentially leveraged for human benefit—is secured by a very small population of reproductive individuals. Such a model system would, in turn, be expected to exhibit sensitivity to changes in per individual reproductive rates.

Although there is a rich honey bee literature there are few published mathematical models which can be used to quantify and test hypotheses about how hives fail, and even fewer attempting to address CCD specifically. Existing honey bee models can be lumped into two broad categories: (1) detailed empirically based simulation models designed for a specific purpose (for example, [14, 15, 16]; but see also [17] for a discussion of how a “new integrated model could be built” through reformulating existing constructs), and (2) simpler differential equation models with few state variables which we view as more amenable to hypothesis testing (for example, [18, 19, 20, 21, 22, 23]).

One potentially crucial factor in hive survival is the prospect of an Allee effect. An Allee effect is defined by the per-individual (or per unit abundance) growth rate at low population abundances being an increasing function of abundance [24, 25, 26, 27, 28]. An Allee effect is practically an ecological tenant in obligate social animals in which reproduction, or survival, or both are enhanced by greater numbers [29]. Numerous examples of Allee effects in many plant, animal, and microbial species have been documented, due to many mechanisms by which increased population abundance can contribute to increased fitness; see reviews, [30, 31, 32]. In particular, Allee effects in ants have been documented with experimental manipulations of ant colonies [33], the results of which inspire a strong suspicion that Allee effects influence other eusocial insects as well.

Few honey bee models published so far have included an Allee effect as a potential factor in hive dynamics. The stage-structured model of Kribs-Zaleta and Mitchell [23] casts CCD as caused by a transmissible infection brought to the hive by foragers (older adults) that instigates a lack of adequate hive bees (younger adults). The stage imbalance creates an Allee effect from the lack of care of the brood and hive. The stage-structured model of Khoury *et al*. [20] contains an eclosion rate (recruitment to hive worker stage) that is an increasing function of the number of adult bees, but an increasing recruitment rate per se does not constitute an Allee effect.

Here we construct a mathematical model to quantify the loss of resilience in collapsing honey bee colonies due to the presence of a strong Allee effect. We propose that a strong Allee effect in the growth dynamics of honeybees is the major ultimate cause of hive failure in general, and the phenomenon of CCD in particular. The model is a deterministic, one-state-variable model that formulates the recruitment and loss rates of hive members in terms of density dependent social components. The presence and magnitudes of a lower unstable equilibrium (critical hive size) and an upper stable equilibrium for the number of adult bees in a hive depend on the values of model parameters. When the values of parameters change, the locations and existence of the equilibria can change in ways that place a hive in jeopardy. The model gives predictions about the likely effects on hive numbers of changing different environmental factors that are amenable to testing. We intend for the model to provide a framework for the design of needed empirical investigations into the joint and separate causes of the collapse of honey bee hives.

Before we present the model, some discussion of the terminology surrounding Allee effects is warranted. We follow the distinction made by recent authors between a strong (or critical) Allee effect and a weak (noncritical) Allee effect: a strong Allee effect refers to a population that exhibits a "critical size or density" below which a population declines to extinction and above which it survives, while a weak Allee effect refers to a population that lacks a critical density, but where, at lower densities, the per-individual growth rate is everywhere positive but still rises with increasing densities [34, 30]. The same distinction was made earlier by Clark [35], under the terms "critical depensation" and "noncritical depensation". However, we are not fond of the term "demographic Allee effect", coined by Stephens *et al*. [34] to describe any net increase in per-individual growth rate at low population abundances. The term unfortunately risks confusion with demographic stochasticity as well as the demographically stochastic models of Lande [36] which were claimed to have an Allee-like behavior and which were a particular target of Stephens *et al*.'s critique. We see no reason to affix the word "demographic" to the concept known universally beforehand simply as an Allee effect. In cases where confusion is possible, we suggest instead the term "net Allee effect". Stephens *et al*.'s real point was that an Allee effect could be caused by different density dependent mechanisms operating on recruitment (or births), or mortality, or both. Stephens *et al*. [34] termed such mechanisms "component Allee effects", a distinction we follow and which is worth perpetuating in terminology.

### Honey Bee Model

Honey bee hives have some control over the ratios of development stages in the hives [20, 22]. Adult bees have two basic stages: the younger adults work in the hive, and the older adults forage for food. If too few foragers are present in a hive, the promotion of hive bees to foragers is accelerated, while if too few hive bees are present, foragers can revert to hive roles. The phenomenon of stage ratio adjustment suggests that a model with just one state variable, namely number of adult bees, might capture salient aspects of the dynamics of a hive's numbers.

### Asocial hive model

Let *N*_*t*_ be the number of adult bees of all stages in a hive at time *t*. Consider first a model with a complete absence of social effects of adult bees on recruitment and mortality. Gains to *N*_*t*_ are in the form of brood production by the queen. The maximum egg-laying rate of the queen would result at best in a constant rate of recruitment for *N*_*t*_. Losses from *N*_*t*_ occur through mortality. The rate of such losses in the absence of social effects would be expected to be proportional. The net rate of change in the variable *N*_*t*_ is the rate of gains minus the rate of losses, or recruitment − mortality. The model of the number of adult bees in an asocial hive becomes

$$\frac{dN_{t}}{dt}=\;\lambda \;-\;\mu N_{t}.$$ (1)

Here *λ* is the constant rate of recruitment of adult bees, and *μ* is the constant per-individual rate of mortality. The course of population increase from an initial value *N*_0_ can be calculated from the integrated form of the model:

$$N_{t}=\;\frac{\lambda }{\mu }+\;\left(N_{0}-\;\frac{\lambda }{\mu }\right)e^{-\mu t}.$$ (2)

**Fig 1:**
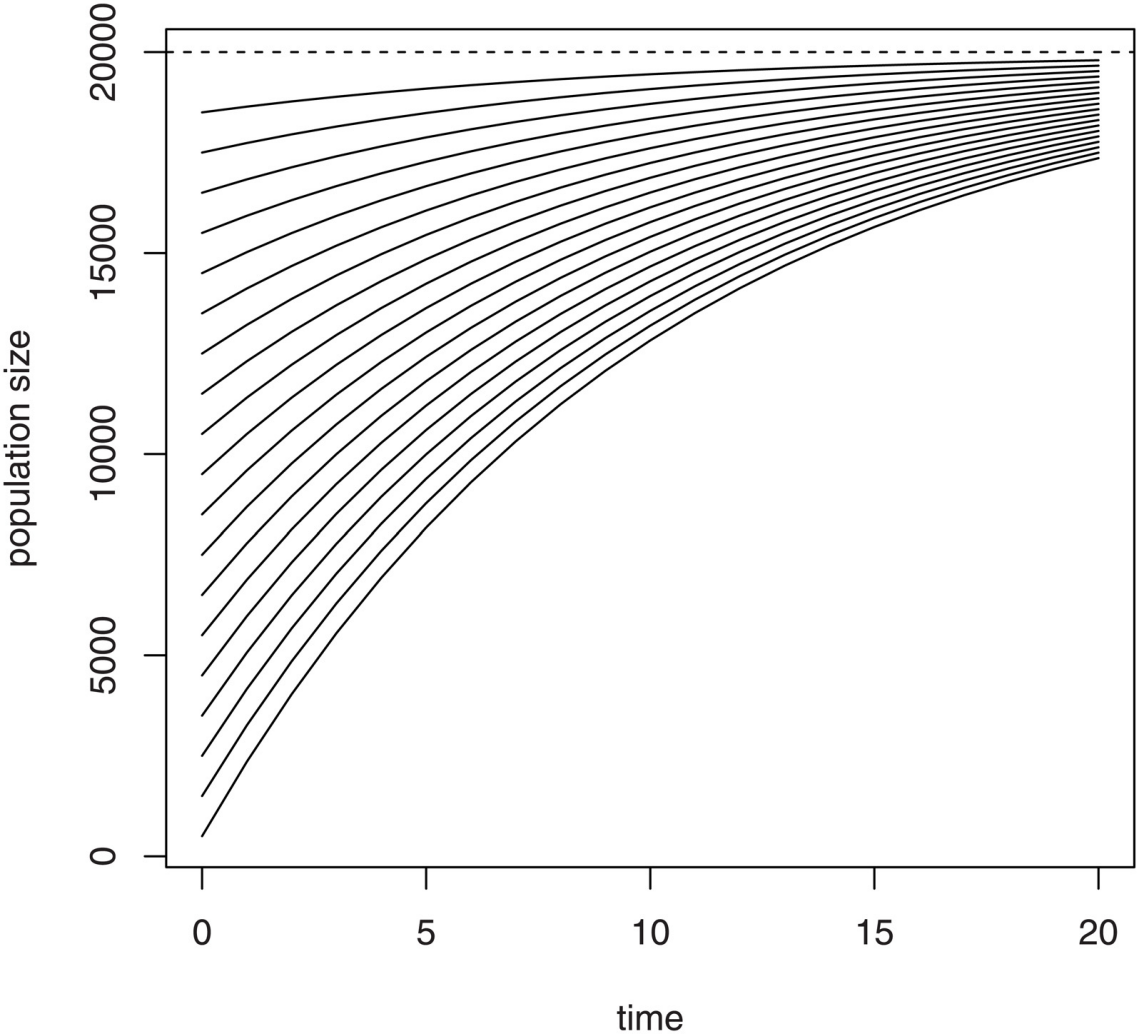
Asocial model solutions. Solution trajectories for number of adult honey bees in a hive (population size, vertical axis) for different initial bee numbers under the asocial model of hive growth in which the adult recruitment rate and the per-individual mortality rate do not depend on population size.

The asocial model (Eqs 1 or 2) predicts that an initially small value of *N*_*t*_ at first will increase rapidly but will eventually approach an equilibrium size of *λ*/*μ* (Fig 1). Normally, however, a hive would throw off a swarm before reaching such an equilibrium. Note that the numbers of bees in an individual hive cannot grow exponentially, because the queen has a maximum possible rate of brood production. A bee population in a region, on the other hand, can increase exponentially by hive-splitting and swarming.

### Social hive model

Various aspects of bee biology contradict the above asocial model and suggest that both the recruitment rate and the mortality rate are affected by *N*_*t*_. Most components of hive function require the social and cooperative behavior of adult worker bees. Adult bees are involved in caring for and feeding the queen, caring for and rearing the brood, foraging cooperatively for food, hive temperature control, hive cleaning, and hive protection.

One crucial fact is that a queen cannot establish a hive without a swarm of sufficient size. The numerical challenges to hive viability are well-known to beekeepers, and commercial packages for establishing a hive average around 11,000 bees [37]. Contrarily, the asocial model predicts that bee numbers will increase for all positive values of *N*_0_ below the equilibrium (Fig 1).

**Fig 2:**
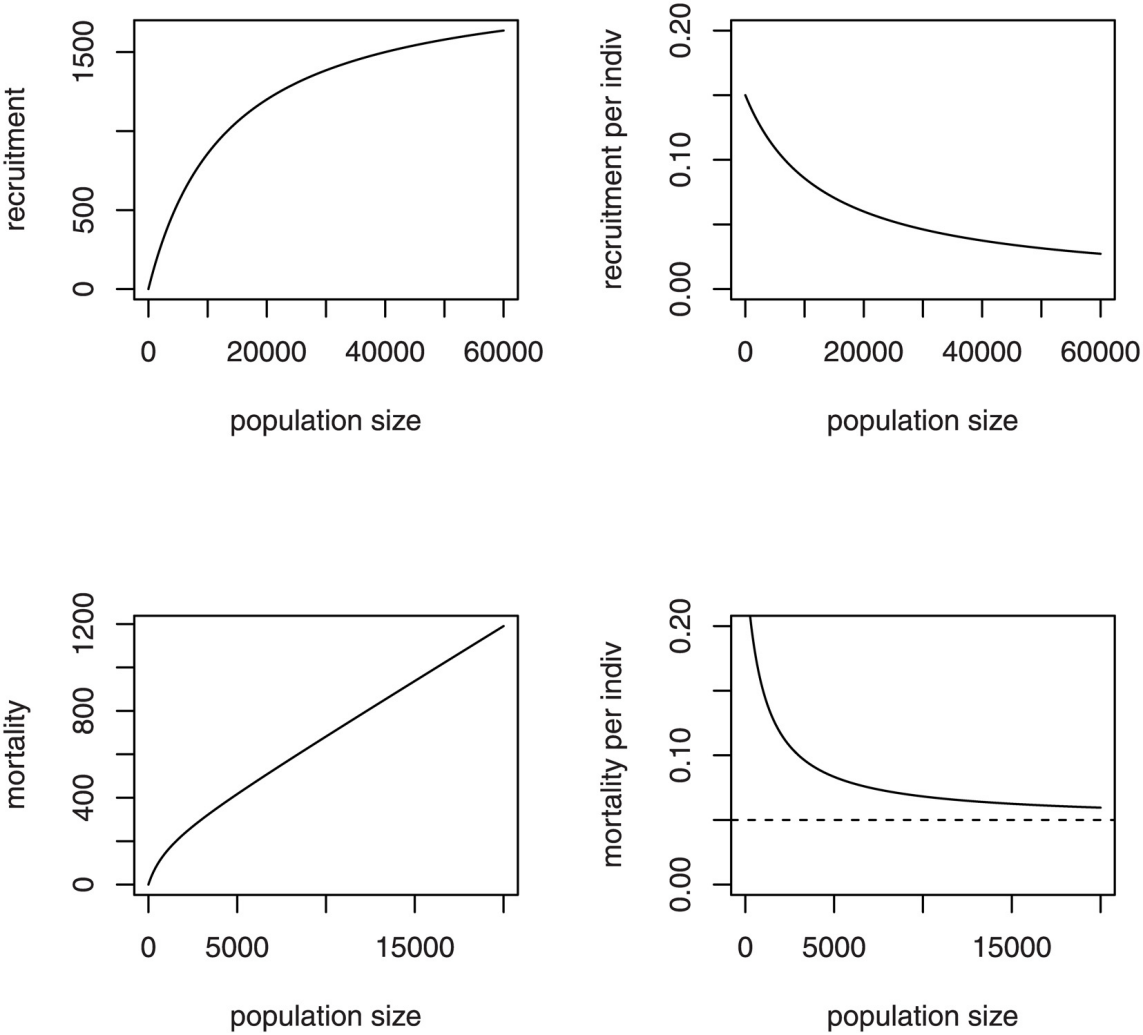
Rate functions in the social hive model. Adult recruitment rate (upper left), per-individual adult recruitment rate (upper right), adult mortality rate (lower left), and per-individual adult mortality rate (lower right) as functions of adult bee numbers (population size) for the social model of honey bee hive growth.

We assume that the production of viable recruits of adult bees would decrease if *N*_*t*_ becomes smaller, because adult bees must feed and care for the brood and the queen. By regarding the variable *N*_*t*_ basically as a surrogate for food supply for brood members, we surmise that the recruitment rate function can be formulated as:

$$\text{recruitment}=\;\frac{\lambda N_{t}}{\theta +\;N_{t}},$$ (3)

where *λ* and *θ* are positive constants. The expression is in the form of a functional response equation from predator-prey theory and approaches an upper maximum, *λ*, as *N*_*t*_ becomes large (Fig 2, upper left). The constant *θ* is an inverse measure of cooperative function in recruitment and is the number of bees at which recruitment is half of maximum. As *θ* becomes larger, the recruitment rate approaches *λ* more slowly. The per-individual recruitment rate is a concave-up, decreasing function with intercept at *λ*/*θ* and a lower asymptote of zero (Fig 2, upper right).

We assume also that the per-individual mortality rate decreases as *N*_*t*_ becomes larger, because of the synergies of cooperative foraging, hive maintenance, and hive protection. Certainly, for substantially larger values of *N*_*t*_ the per-individual mortality rate would be expected to increase due to resource and disease issues, but we assume that a hive will send off a swarm well before such numbers are attained. If *μ* is the lower bound to the per-individual mortality rate for the range of values of *N*_*t*_ seen in hives, and *μ*+*α* is the upper bound to the per-individual mortality rate for small values of *N*_*t*_, we model the functional change of the mortality rate by

$$\text{mortality}=\;\left(\frac{\alpha \beta }{\beta +N_{t}}+\;\mu \right)N_{t},$$ (4)

where *α*, *β*, and *μ* are positive constants. As *N*_*t*_ becomes large, the losses to the hive from mortality approach the simple proportional expression *μN*_*t*_ (Fig 2, lower left). The per-individual mortality rate is a concave-up, decreasing function with intercept at *μ*+*α* and a lower asymptote at *μ* (Fig 2, lower right). The constant *β* is an inverse measure of the social effects of bee numbers on mortality and represents the value of *N*_*t*_ at which mortality has fallen halfway to *μ* from *μ*+*α*. A smaller value of *β* corresponds to a more rapid decrease in the per-individual mortality rate.

From the above formulations, our social model of hive numbers is

$$\frac{dN_{t}}{dt}=\;\frac{\lambda N_{t}}{\theta +\;N_{t}}-\;\left(\frac{\alpha \beta }{\beta +N_{t}}+\;\mu \right)N_{t}.$$ (5)

The per-individual net rate of change becomes

$$\frac{1}{N_{t}}\frac{dN_{t}}{dt}=\;\frac{\lambda }{\theta +\;N_{t}}-\;\left(\frac{\alpha \beta }{\beta +N_{t}}+\;\mu \right).$$ (6)

If the rates are such that only small amounts of births or deaths are expected in 1 day, the model can be conveniently recast in discrete time, with the unit of time being 1 day:

$$N_{t+1}\;=\;N_{t}+\;\frac{\lambda N_{t}}{\theta +\;N_{t}}-\;\left(\frac{\alpha \beta }{\beta +N_{t}}+\;\mu \right)N_{t}.$$ (7)

However, this discrete-time version can show erratic or absurd dynamical behavior (such as negative values of *N*_*t*_) if the daily rates are large.

**Fig 3:**
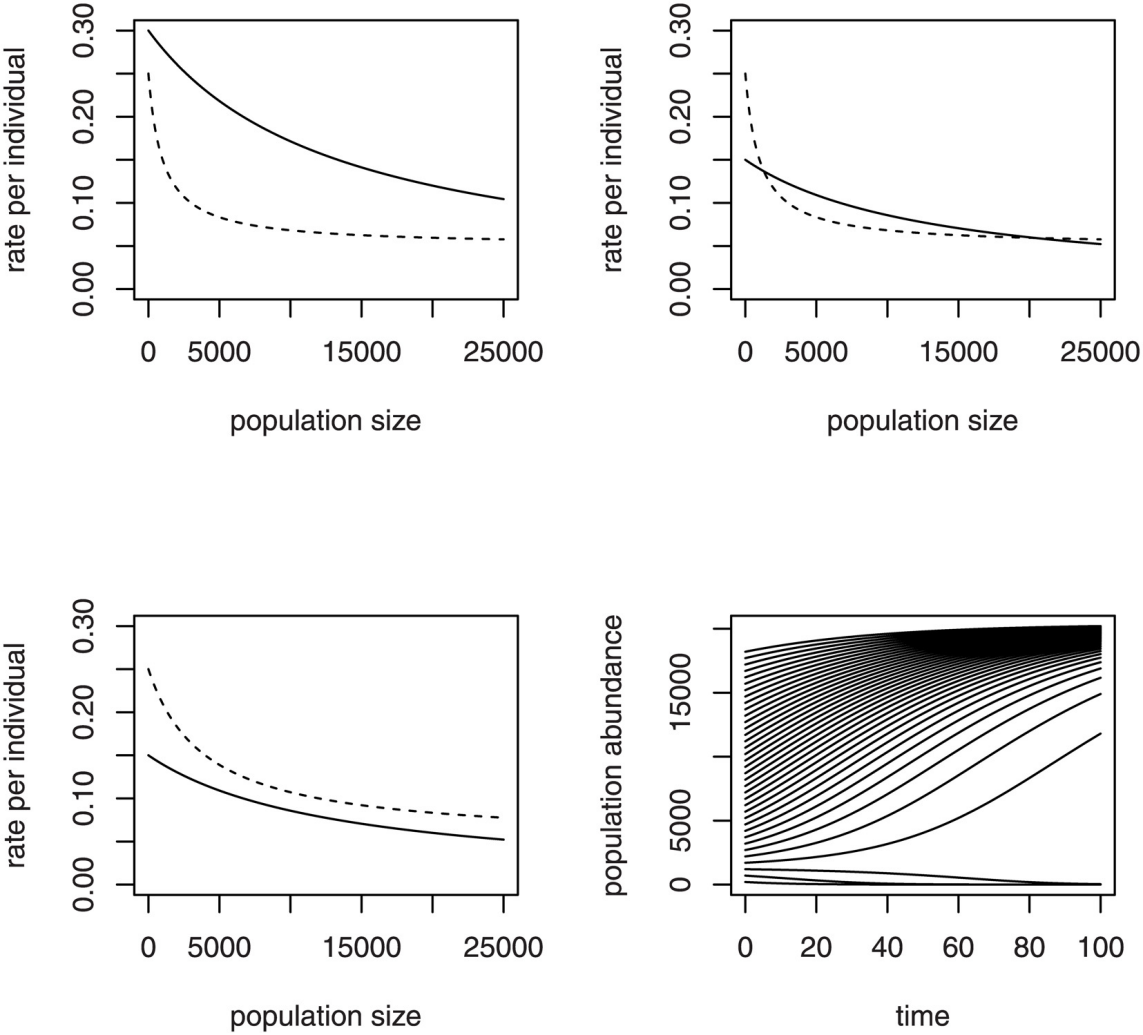
Equilibria and solutions of the social hive model. Three different configurations of the per-individual recruitment rate (solid curve) and per-individual mortality rate (dashed curve) for the social model of hive growth produce different equilibria. Case 1 (upper left): recruitment vertical-axis intercept greater than that of mortality, with one positive and stable equilibrium at the intersection of the curves (at a population size beyond right limit of graph). Case 2 (upper right): mortality intercept greater than that of recruitment with two positive population sizes at which the curves intersect, representing a lower unstable equilibrium in addition to the upper stable equilibrium. Case 3 (lower left): mortality everywhere greater than recruitment, with no positive equilibria. Lower right: solution trajectories for Case 2.

The dynamic behavior of solution trajectories of the social hive model (Eq 5) depend on parameter values. The nonlinear model has no analytical solution formula for *N*_*t*_; the differential equation (Eq 5) must be solved numerically. However, the solution behavior can be discerned easily from the growth rate functions. Three possible configurations of the per-individual recruitment and mortality rates (as in Eq 6) produce three different dynamic cases. Case 1 occurs when the per-individual recruitment intercept *λ*/*θ* exceeds the per-individual mortality intercept *α*+*μ* (Fig 3, upper left). One positive equilibrium exists at the value of *N*_*t*_ where the curves cross, representing a population size at which the net rate of change is zero. The equilibrium is a stable one, which attracts all model trajectories. In particular, all values of *N*_*t*_ below the equilibrium produce a positive net growth rate, and the hive size increases. Case 1 likely does not occur naturally, for it suggests like the asocial model that all hive sizes are viable. In Case 2, the recruitment intercept *λ*/*θ* is below the mortality intercept *α*+*μ* but above the mortality asymptote *μ*, and mortality falls off fast enough to intersect recruitment twice (Fig 3, upper right). The intersections represent two positive equilibria, a lower unstable critical size and an upper stable size. In Case 3, mortality is everywhere greater than recruitment (Fig 3, lower left), which occurs if mortality does not fall off fast enough to intersect recruitment, or simply if *λ*/*θ* is below *μ*. No viable hive sizes exist under Case 3; all model trajectories decrease and converge to zero. In Fig 3 (lower right), numerical solutions of Eq 5 under Case 2 show model trajectories locally diverging from the lower critical size and locally converging to the upper stable size.

We hypothesize that Case 2 represents the functioning of a hive under ordinary viable conditions. As long as hive numbers are above a critical size, the hive thrives and grows in numbers, until the growth slows down and hive numbers move toward an upper stable equilibrium. At some point, before attaining the equilibrium, the hive will split and send out a daughter swarm of sufficient size to be viable to colonize a new area, while retaining sufficient numbers for its own viability.

### Allee effect

**Fig 4:**
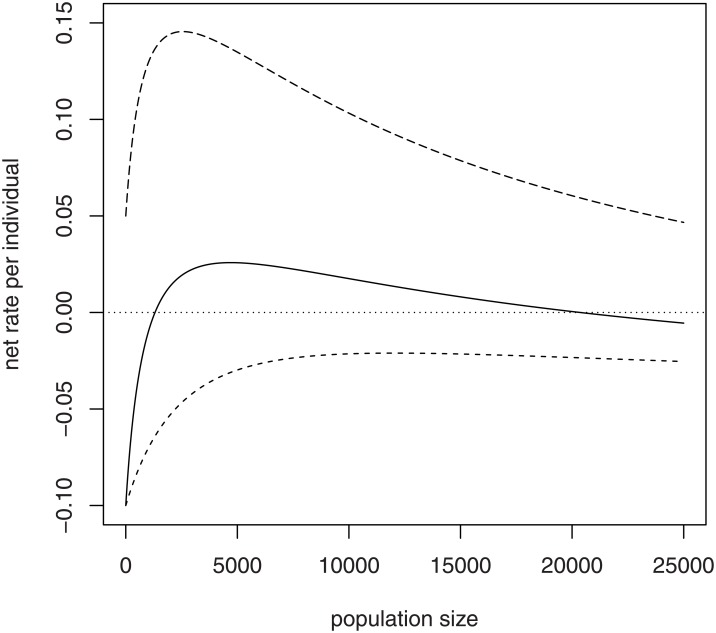
Allee effect in the social hive model. Net per-individual growth rate for the social model of hive growth, plotted for Case 1 (upper curve, long dashes), Case 2 (middle curve, solid) and Case 3 (lower curve, short dashes), with cases as defined in Fig 3.

In all three cases, the net per individual growth rate (Eq 6) is initially an increasing function of population size (Fig 4). An increasing per-individual net growth rate at low population sizes is the hallmark of an Allee effect. In Case 2, the net per individual growth rate commences initially below zero, rising to become positive before turning to decrease back across zero again (Fig 4, solid curve). The range of *N*_*t*_ values below the first zero crossing correspond to negative net per-individual growth rates and represent population sizes that are too small for a hive to become established. The range of *N*_*t*_ values between the two zero crossings represent population sizes at which net per-individual growth rate is positive; a hive can become established. The lower zero crossing is the lower unstable equilibrium, the critical population size below which the hive is not viable and population collapse occurs. The upper zero crossing is the upper stable equilibrium at which the mortality rate overtakes the recruitment rate. Under favorable conditions a hive would be expected to send out a swarm at or near this stable size. Case 1 (Fig 4, long-dashed curve) displays a weak Allee effect in which the net per individual growth rate has an increasing section but is not small enough at low *N*_*t*_ to be negative. Population growth would be positive but sluggish at low population sizes. Case 3 (Fig 4, short-dashed curve) is a situation in which an Allee effect is present but the net per individual growth rate is everywhere negative. The population would decline at all values of *N*_*t*_.

### Hive collapse

Equilibria in the model are found by setting the expression for *dN*_*t*_/*dt* in Eq 5 equal to zero and solving for values of *N*_*t*_ that satisfy the resulting equation. We denote equilibria by $\overset{-}{N}$. Clearly ${\overset{-}{N}}_{0}\;=\;0$ is one such equilibrium that always exists. Two other potential equilibria are roots to a quadratic equation given by *aN*^2^+*bN*+*c* = 0, where *a* = −*μ*, *b* = *λ*−*αβ*−*μθ*−*μβ*, and *c* = *β*(*λ*−*αθ*−*μθ*). The roots are

$${\bar{N}}_{1}=\;\frac{-b-\;\sqrt{b^{2}-4ac}}{2a},$$ (8)

$${\bar{N}}_{2}=\;\frac{-b+\;\sqrt{b^{2}-4ac}}{2a}.$$ (9)

The roots are real-valued if *b*^2^−4*ac*>0, which written out becomes

$$\frac{{\left(\lambda -\alpha \beta -\mu \theta -\mu \beta \right)}^{2}}{\mu \beta \left(\mu \theta +\alpha \theta -\lambda \right)}>1.$$ (10)

When the roots are real, they will both be positive and correspond to Case 2 (Fig 3) when the lower root is positive. Setting ${\overset{-}{N}}_{1}>0$ yields

$$\frac{\lambda }{\theta }\;<\;\alpha +\;\mu ,$$ (11)

or just the condition that the intercept of the per-individual recruitment rate exceeds that of the per-individual mortality rate.

**Fig 5:**
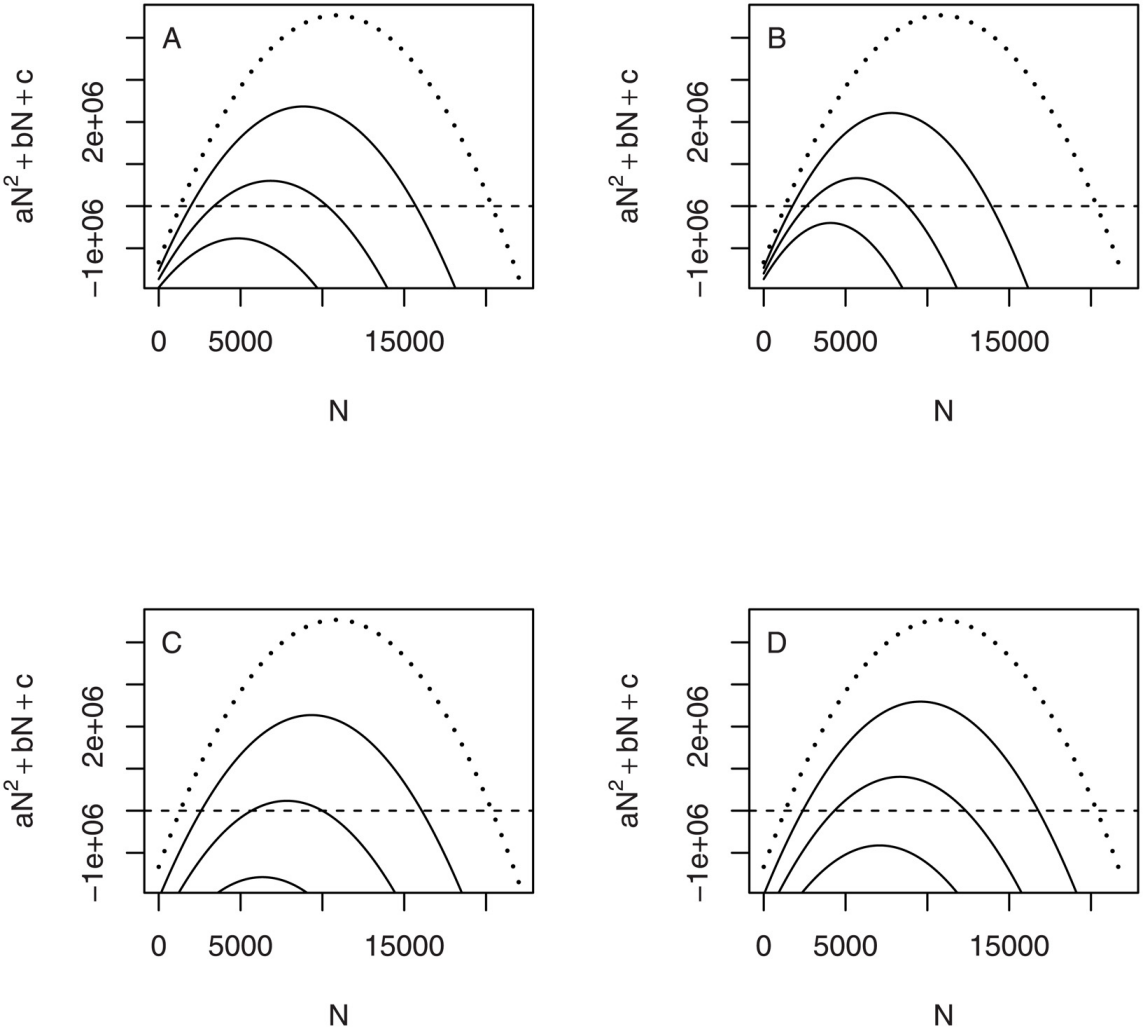
Effects of parameter changes on equilibria in the social hive model. Equilibria of the social model of hive growth are roots of a quadratic equation involving the model parameters *λ*, *θ*, *β*, *α*, and *μ*. Roots if they exist are shown as intersections between quadratic curve (solid curve) and zero (dashed line). Each panel shows the quadratic for three different values of a parameter. Height of the quadratic decreases as: (A) *λ* decreases, (B) *μ* (and/or *α*) increases, (C) *θ* increases, (D) *β* increases. Decreasing height of the quadratic is accompanied by the lower root increasing and the upper root decreasing, until the two roots merge and vanish (lowest quadratic, A-D).

The left side of the discriminant inequality (Eq 10) contains all the model parameters and suggests that colony collapse can result from numerous causes. Any parameter change that causes the discriminant quantity to decrease closer to 1 (or causes *b*^2^−4*ac* to decrease closer to 0) in turn causes the two roots ${\overset{-}{N}}_{1}$ and ${\overset{-}{N}}_{2}$ to approach each other (Fig 5). Such a parameter change in this model causes the lower unstable equilibrium ${\overset{-}{N}}_{1}$ to increase and the upper stable equilibrium ${\overset{-}{N}}_{2}$ to decrease. A colony of formerly viable size could find itself below the increased critical size ${\overset{-}{N}}_{1}$ and collapse would ensue. A colony near a stable equilibrium ${\overset{-}{N}}_{2}$ of diminished size could find itself unable to send off a viable swarm without endangering its own viability. When the discriminant quantity is reduced below 1, the two real-valued equilibria merge and disappear into the complex plane. As a result, no viable hive size would exist; a hive of any size would collapse. Note that the discriminant quantity (left side of Eq 10) is an increasing function of *λ*, and a decreasing function of *θ*, *β*, *α*, and *μ*. Any environmental factor that either (A) decreases the maximum egg laying rate (Fig 5A), (B) decreases the social enhancement of recruitment (Fig 5B), (C) decreases the social amelioration of mortality (Fig 5C), or (D) increases the maximum or minimum mortality rates, singly or in combinations (Fig 5D), could trigger a colony collapse.

### Ecological resilience

Ecologists have been studying the prospect that some ecological systems could have alternative stable states, and that "regime shifts" could occur as a result of disturbance [38, 39]. The alternative stable states of a honey bee hive are an upper equilibrium size and zero. A healthy hive persists, meaning that the upper stable state has a wide and deep "basin of attraction" that makes hive numbers resistant to perturbations. Swarming produces two smaller hives that are temporarily at greater risk of being pushed below the lower critical size by an unlucky series of environmental shocks, but the wide and deep basin rapidly restores each hive to the upper stable state.

Ecologists often write about stability in terms of a "marble in a bowl" analogy [40]. A marble rolls to the bottom of a bowl, eventually coming to rest at the lowest point. In a one-state-variable model the bowl is mathematically defined by a potential function, which we denote by *u*(*N*). If the rate of change of a state variable is given by

$$\frac{dN_{t}}{dt}=m\left(N_{t}\right),$$ (12)

where *m*(*N*) is a rate function, the potential function is given by

$$u^{\prime }\left(N\right)=-m\left(N\right)$$ (13)

or

u(N)= −∫m(N)dN. (14)

The height of the function *u*(*N*) is determined only up to an arbitrary constant, but the local slope of *u*(*N*) is the speed with which the system state is changing, the change being in the downhill direction toward an attracting state. For the social hive model (Eq 5) the potential function is

$$u\left(N\right)=\;\left(\alpha \beta -\lambda \right)N+\;\frac{\mu }{2}N^{2}\;-\;\alpha \beta^{2}\text{log}\left(\beta +N\right)+\;\lambda \theta \text{log}\left(\theta +N\right).$$ (15)

**Fig 6:**
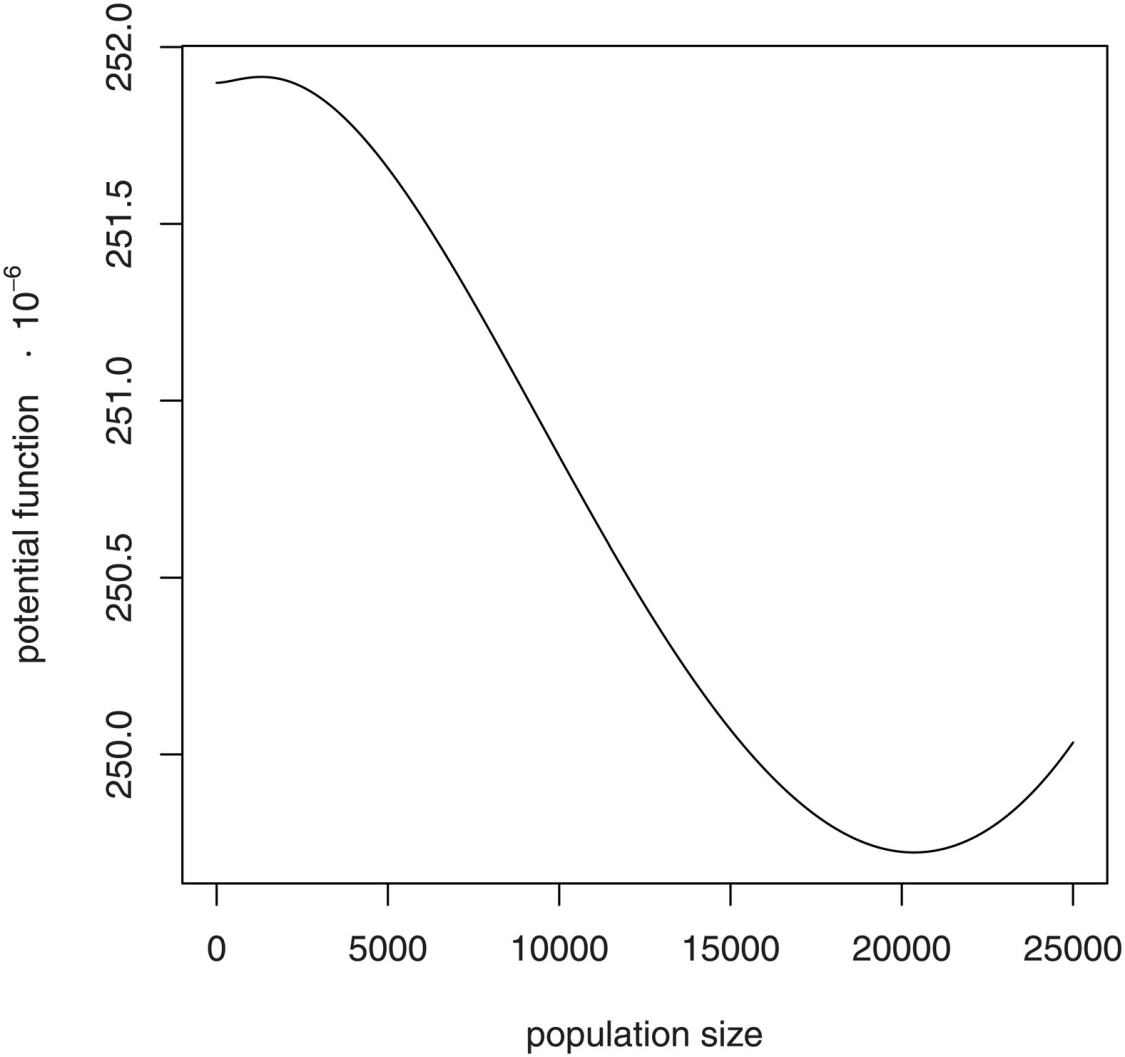
Potential function for the social hive model. The potential function is depicted under a strong Allee effect with upper stable equilibrium (as in Fig 3, Case 2). Basin of attraction to the upper stable equilibrium is deep and wide, conferring resistance to perturbations that could reduce population size and therefore move the system to the high, shallow basin of attraction to zero.

The potential function for the social hive model (Eq 15) under a strong Allee effect (Case 2) has two local minima, representing the two stable states of zero and the upper equilibrium (Fig 6). The success and persistence of hives under ordinary conditions suggests that the basin of attraction to the upper stable equilibrium is steep, deep, and wide. These properties would confer resistance to stochastic shocks that could perturb the hive from the upper stable basin into the collapse basin. Also, the basin of attraction to zero is flatter, shallow and narrow. In this collapse basin, approach to zero is slow and potentially corrected by random, favorable demographic events.

"Resilience" of an ecological system is frequently defined as the resistance of a system to perturbations that would shift the state of the system from one basin of attraction into the basin of attraction for an alternative stable state [41, 42]. Such resistance is determined by the properties of the basin of attraction in question. Potential functions per se generally do not exist for multiple variable systems, but basins of attraction do exist. For one-variable systems, potential functions form a useful graphical representation of the resilience concept. Width, steepness, and depth of a potential function basin contribute to resilience of that stable state.

**Fig 7:**
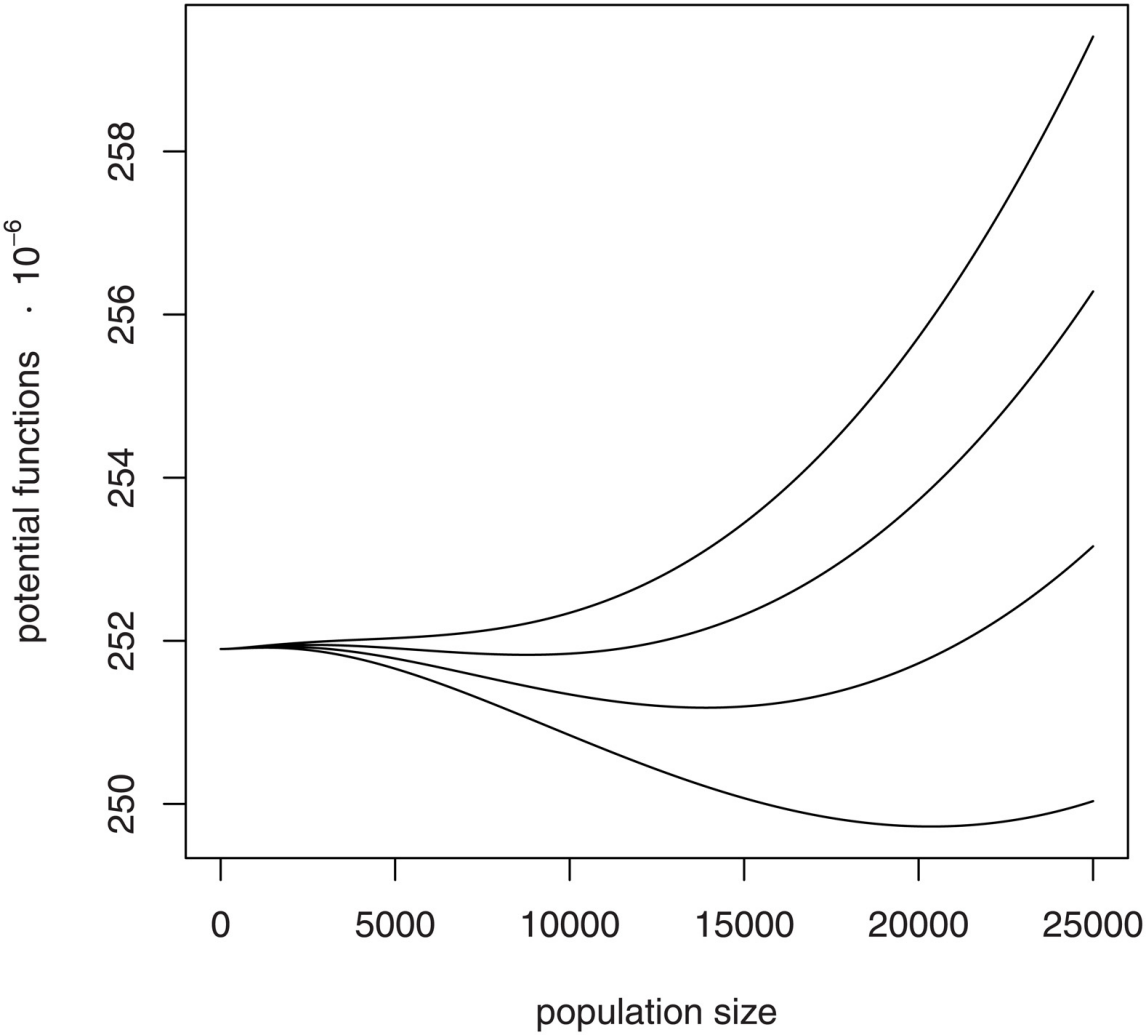
Effects of parameter changes on the social hive potential function. The potential function for the social model of hive growth is depicted involving unfavorable changes in the model parameters *λ*, *θ*, *β*, *α*, or *μ*. Basin of attraction to the upper stable equilibrium becomes shallower and narrower and eventually disappears altogether.

The potential function of the social hive model (Eq 15) reveals a severe loss of resilience in response to unfavorable parameter changes (Fig 7). Decreases in *λ*, or increases in *θ*, *β*, *α*, or *μ*, cause the basin of attraction to the upper stable equilibrium to become shallower and narrower. With continued degradation of hive parameters, the basin of attraction to the upper stable equilibrium eventually disappears altogether.

## Discussion

### Allee effect in honeybees

Early in ecologists' discourse relating to negative density dependence and logistic-style population growth models, various investigators pointed out that a variety of biological mechanisms might be expected to produce positive density dependence, at least at low population abundances [24, 25, 26, 43, 27, 44]; see [28] for a review of the early literature on Allee effects. Following the advent of conservation biology as an organized discipline, various population models with Allee effects have been rediscovered and re-reviewed [45, 46]. More importantly, a large catalogue of ecological cases in which Allee effects have been either demonstrated or strongly suspected is accumulating [30, 31, 32, 47].

In the hive model presented here, both recruitment and mortality have component Allee effects. Furthermore, our hypothesis is that the component Allee effects are strong enough for the net rate of production of adult bees to be negative at very low population sizes. Under ordinary environmental conditions, a queen cannot establish a hive alone, nor can a queen establish a hive with a swarm that is too small. Such a critical Allee effect is normally not a significant factor in a regional bee population with abundant wild hives sending off viable swarms, and with domestic hives being propagated well above criticality through attentive beekeeping—initiating hives with “package bees” containing worker numbers generally sufficient for colony establishment under a wide range of resource conditions, combining hives when population levels become low, and less frequently, splitting hives when high.

However, environmental conditions, both natural and anthropogenic, can cause various changes in parameter values (Fig 5) that can result in the increase of the critical hive size. With increased mortality rate (*μ* or *α*), or some degradation of cooperative functions (increased *θ* or *β*), an ordinarily viable hive could find itself on the wrong side of the critical hive size. Such mortality increase or degradation of cooperative functions lowers the stable equilibrium size as well, so that the bees could achieve a stasis at a size that is too small for sending off a viable swarm and yet is dangerously close to the critical size. A low stable size subjects the hive to greater risks from ordinary stochastic environmental events that might push the hive below the critical size [28, 48]. Further degradation of conditions could be enough to merge the critical size with the stable equilibrium size, producing Case 3 (Fig 3), resulting in hive collapse at all population sizes.

### Loss of resilience

Our model suggests that hive collapse in general, and CCD in particular, can have many possible causes. Indeed, biologists have not yet been able to implicate a single environmental factor accounting for even a majority of instances of CCD [49]. Recent reviews of the burgeoning literature on CCD [4, 5] give an emerging picture of multiple possible proximate causes, each working singly or jointly, including toxins such as pesticides and other xenobiotics, *Nosema* sp fungal infection, *Varroa* sp. mites, poor nutrition (possibly creating adverse changes in the bee gut microbiome), and various viruses such as Israeli Acute Paralytic Virus. Global climate change could exacerbate any or all of the proximate causes. Such environmental factors could potentially stress hives at multiple vulnerable points of bee biology, such as by reducing communications or foraging abilities, reducing egg laying, increasing stage specific developmental times, increasing mortality, or decreasing cooperative hive protection. These stresses are captured in the model through adverse changes in the egg laying (*λ*) and mortality (*α* and *μ*) parameters and in the recruitment (*θ*) and mortality (*β*) social cooperation parameters.

Our model further suggests that the ultimate cause for the widespread collapse of bee colonies is to be found in the strong Allee effect produced by the evolution of eusociality in bees [50], and perhaps more generally in Hymenoptera. In a pre-*Varroa* study conducted in Louisiana, USA, involving starting populations ranging from 2,300–35,000, Harbo [51], demonstrated a strong positive relationship between the gain of brood and honey (mg/bee/day) and initial colony population levels. Furthermore, Harbo suggested that the decline in brood production observed in the smallest colonies “may not reflect a tendency to produce less brood, but rather a physical inability to produce more brood.”

More recently, a pair of studies [33, 52] presented what may be the first evidence of Allee effects in other eusocial insects. In controlled experiments conducted with the invasive Argentine ant (*Linepithema humile*), Luque *et al*. [33], demonstrated a positive feedback between worker and queen numbers, which they posit may have contributed to the evolution of large colony sizes (see also [53]). In addition, Bryden *et al*. [52] performed controlled experiments with bumblebee (*Bombus terrestris*) colonies and demonstrated that sublethal doses of a neonicotenoid pesticide could impair colony function and cause eventual colony collapse, consistent with a mathematical model of colony dynamics containing positive density dependence. Although the in-colony dynamics of both bumblebees and ants differ from those of the honey bee, the consequence of the tight social organization in honey bees is the likelihood that even unstressed colonies have a meaningful critical size (strong Allee effect), while the bumblebee and ant examples represent weak Allee effects potentially exacerbated into strong Allee effects in the presence of environmental stressors. In any event, accumulating evidence suggests that the highly organized social structures in eusocial insects are associated with differing reproductive rates at differing colony sizes. The evidence argues for a more comprehensive framework for studying colony dynamics, one that includes the existence of component Allee effects [33], along with the consideration for castes and the division of labor [20, 21].

In the numerous hypothesized reasons for colony collapse, a strong Allee effect is the veritable elephant in the room; the widespread occurrence of colony collapse seems to be characterized by a threshold or tipping point in dynamical behavior. Concurrently, even healthy feral or domestic hives need sufficient adult bee numbers to be viable, pointing to a low but ordinarily attainable minimum hive size for viability. The critical hive size, however, is sensitive to various factors that are likely traceable to environmental change. Reduced resistance to pathogens, or disruptions to social behavior or communication, could be caused by xenobiotics, climate change, or both. An underappreciated but key understanding about Allee effects to emerge from modeling is that adding impacts to a population such as increased mortality or harvesting not only just reduces population size but also makes the Allee effect more severe [28]. The critical Allee size increases, the carrying capacity decreases, the sluggishness of growth at low population sizes becomes more sluggish, the risks of stochasticity are increased.

For the honey bee, a strong Allee effect and environmental stressors combine to produce a net loss of resilience in hive function. Certainly, both natural history and stochastic modeling strongly assert that zero is the ultimate state of any population. Successful populations are resilient: they are able to resist extinction for long time periods. For a honey bee hive, the competitive success conferred by extreme sociality may be turning to failure as a result of environmental changes taking place across the contemporary landscape. Every small impact to hive function fills in the basin of persistence a little more (Fig 7), with the shallower basin then rendering the hive's existence even more precarious.

Preliminary experiments are starting to untangle the complex phenomenon of CCD. For example, a widely noted recent experiment of Lu *et al*. [54] implicated sub-lethal doses of neonicitinoid pesticides as a major contributor to CCD. However, the small sample sizes and limited design layout used by Lu *et al*. [54] suggest the need for further expanded experiments to estimate interactions with other environmental factors as well as more detailed dose responses. Also, in a 3 yr experiment, Dively et al. [55] tested the sub lethal effects of imidacloprid residues on honey bee colonies. The imidacloprid exposure, in varying doses, affected winter colony survival, rates of queen failure and broodless periods, and rates of *Varroa* infestation, suggestive of the chronic resilience loss predicted by our model. More experiments initiating hives with differing initial numbers of bees, under control and treatment conditions, could help sort out the responses of hive-level properties such as equilibria and hive growth rates to factors suspected of being CCD causes. Dynamic models such as the one presented here can help in the design of experimental layouts and in the quantification of effect sizes.

## Concluding Remarks

The prospect of continued decline of vital pollination services of honey bees is alarming. Management practices for honey bees must face the likely necessity of compensating for a strong Allee effect made worse by the “regime shift” [38, 39] resulting from the arrival of *Varroa* in North America, the compounding effects of 21^st^ Century cropping systems, and changes in climate. Much will be gained, however, from efforts that contribute significantly to regional scale population viability in the honey bee, such as spatially extensive pesticide “best management practices” for beekeepers and crop producers (for example, North Dakota Pollinator Plan; http://www.nd.gov/ndda/files/resource/NorthDakotaPollinatorPlan2014.pdf) and conservation programs that contribute to the improvement of foraging resources for all pollinator species (for example, the Environmental Quality Incentives Program or EQIP; USDA-NRCS; http://www.nrcs.usda.gov/wps/portal/nrcs/detail/national/programs/financial/eqip/?cid=stelprdb1242633).

And lastly, widespread evidence for Allee effects associated with the population dynamics across many organisms, including honey bees, argues strongly for the adoption of more stringent precautionary principles designed specifically for promoting species’ viabilities. Betting against Allee effects is a losing game.

## Supporting Information

S1 R Scripts R scripts for figures. Text file containing 7 scripts written in the R programming language for reproducing Figs 1–7 of this article.
(TXT)
